# A Case Report of Perforated Appendix Vermiform Attached to the Left Ovary

**DOI:** 10.7759/cureus.51270

**Published:** 2023-12-29

**Authors:** Kaveh Mozafari, Thomos Kang

**Affiliations:** 1 Surgery, West Suburban Medical Center, Oak Park, USA; 2 Medicine, St. George's University School of Medicine, St. George, GRD; 3 General Surgery, Ascension Saint Agnes Hospital, Baltimore, USA

**Keywords:** rare appendix vermiform, complex appendix surgery, gastro-intestinal surgery, left ovary appendix, ovary, perforated appendix vermiform

## Abstract

Acute appendicitis, a typical abdominal emergency, presents in nonperforated and perforated forms. While nonperforated cases often respond well to antibiotics or appendectomy, perforated cases pose more significant challenges. We report a unique case of appendicitis in a 34-year-old female with an unusual anatomical adherence to the left ovary. The patient successfully underwent laparoscopic surgery, during which precise incisions were made to accommodate the unique location of the appendix. This tailored approach contributed to a positive surgical outcome. This case underscores the importance of tailored surgical approaches in managing complex appendiceal variations.

## Introduction

Appendicitis, a common abdominal surgical emergency worldwide, often presents with distinct clinical features. Its lifetime risk is estimated at 8.6% in males and 6.9% in females [1]. This condition is broadly categorized into nonperforated and perforated forms, each with unique clinical characteristics and management approaches. Nonperforated appendicitis, constituting around 80% of cases, lacks clinical or radiographic signs of perforation. In contrast, perforated appendicitis is associated with acutely ill patients, often characterized by dehydration and electrolyte imbalances [2].

Pain localization in appendicitis typically occurs in the right lower quadrant unless intra-abdominal structures restrict the inflammation, leading to generalized discomfort in cases of generalized peritonitis. Imaging studies reveal a spectrum of presentations, including inflammatory masses, phlegmons, and intra-abdominal or pelvic abscesses, with rare scenarios involving retroperitoneal abscess formation and enterocutaneous fistula [3]. The decision-making process for appendectomy involves weighing the benefits of open appendectomy (OA) vs. laparoscopic appendectomy (LA). LA has emerged as the preferred surgical intervention where feasible, offering advantages such as lower infection rates, reduced morbidity, shorter hospital stays, and superior quality of life [4,5].

Understanding the diverse presentations of appendicitis and the evolving surgical landscape is crucial for the effective management of the condition. This report presents a unique case of appendicitis where the appendix was found adherent to the left ovary, challenging conventional anatomical expectations and highlighting the importance of tailored surgical approaches. The attachment of the appendix to the left ovary occurs in less than 0.05% of cases, making it a rare anatomical variation [6]. This report aims to elucidate one such case, underscoring the rarity of this occurrence and the consequent potential for misdiagnosis.

## Case presentation

The patient was a 34-year-old female with a past surgical history of cholecystectomy and sternotomy who presented to the emergency department with the chief complaint of abdominal pain and loss of appetite. The abdominal pain was on the left lower quadrant, with some radiation to the right lower quadrant and back. The patient had complained of a fever for three days before she visited the emergency department.

Her vital signs were within normal limits with a slight temperature elevation (37.6 °C). The physical exam was significant for tenderness to palpation on the left side of the abdomen and left lower back. The patient denied nausea, vomiting, constipation, diarrhea, headache, and change of vision. Her blood lab results showed a white blood count of 9.8 K/uL (normal range: 4.5-11.0 K/uL), red blood count of 5.59 M/uL (normal range: 4.35-5.65 K/uL), hemoglobin of 12.1 g/dL (normal range: 12-16 g/dl), hematocrit of 38.2% (normal range: 36-48%), and platelet count of 236 K/uL (normal range: 140-400 K/uL); other blood chemical lab results were within normal limits.

**Figure 1:**
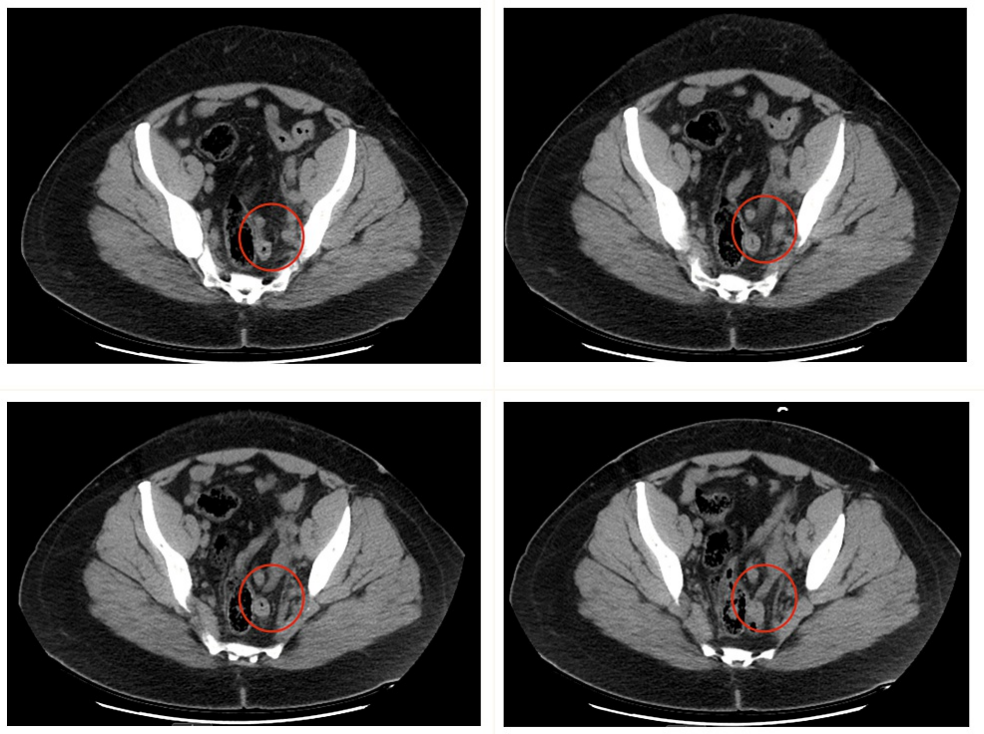
Transverse CT scan depicting appendicitis at different levels The appendix (depicted by the red circle) extends to and is inseparable from the left ovary. There is no adjacent inflammatory change CT: computed tomography

**Figure 2:**
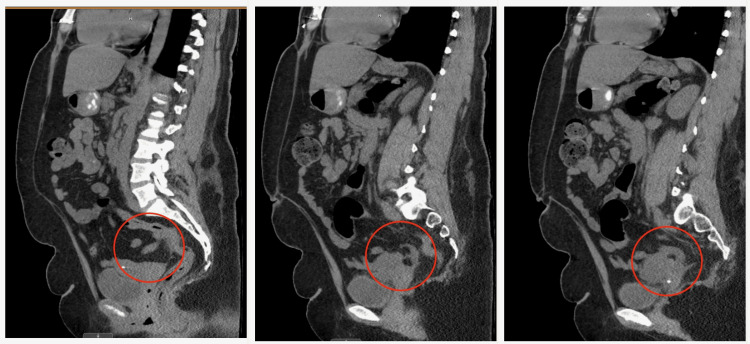
Sagittal CT scan depicting appendicitis The appendix is prominent, measuring up to 12 mm in diameter, possibly due to early appendicitis (illustrated by the red circle) CT: computed tomography

The CT exam revealed a prominent appendix measuring up to 12 mm in diameter, possibly due to early appendicitis (Figures 1-2). There was no adjacent inflammatory change; however, the appendix did extend to and was inseparable from the left ovary. The surgical team decided to proceed with appendectomy after all preop requirements were fulfilled and other differential diagnoses, such as diverticulitis, appendicitis, IBD, and ovarian torsion, were deemed less plausible.

The decision was made to perform a laparoscopic surgery on the patient. During the vertical incision through the umbilicus, an incidental fat-containing umbilical hernia was appreciated, which was subsequently repaired. The terminal ileum was identified based on the Treves fold in the pelvis's midline. There were some adhesions between the uterus and the cecum, and the appendix was observed to be stuck to the left ovary. There was a frankly purulent fluid collection consistent with an abscess, which was suctioned out.

The distal third of the appendix appeared erythematous and affected by the inflammation and the adhesions. While the body and base of the appendix were completely normal, the base of the appendix was in an inferior position, and the appendix was pointing superiorly. The umbilical hernia sac and the appendix were sent to pathology for additional examination. Analysis of the umbilical hernia sac tissue identified adipose tissue, aligning with hernia sac content. In contrast, the appendix specimen showed notable findings of patchy acute appendicitis accompanied by lymphoid hyperplasia. The patient tolerated the procedure well and was shifted to the recovery room in stable condition.

## Discussion

This case challenges the conventional understanding of appendiceal anatomy and emphasizes the importance of considering atypical locations in cases presenting with abdominal pain. The laparoscopic approach not only confirmed the unusual adherence of the appendix to the left ovary but also addressed an incidental umbilical hernia and an associated abscess. This discussion explores the diagnostic intricacies and successful management of this unique case.

Nonperforated appendicitis can be treated with antibiotics and appendectomy. The antibiotic approach has the benefit of avoiding surgery and anesthesia. Also, it is not associated with an increased risk of rupture. However, it is associated with a 10-20% failure rate at 30 days [2], as well as 30-40% and 40-50% recurrence rates at one and five years, respectively. Additionally, there is an increased risk of missed neoplasm if the appendectomy is not performed [7].

Our case adds to the growing body of evidence supporting the benefits of laparoscopic appendectomy in complex situations, especially given that patients with an appendix at odd anatomical locations as in our case are already at increased risk of extended hospital stays. Laparoscopic appendectomy in rare anatomical positions is superior to laparotomy since an extensive open incision is required to reach the appendix [6]. Moreover, laparoscopic appendectomy is associated with a lower incidence of wound infections in comparison to open surgery. Additionally, the laparoscopic approach preserves the immune system, leading to fewer infection-related complications compared to its open surgery counterpart [8,9,10,11,12].

Atypical appendicitis locations attached to the left ovary are sporadic presentations, accounting for less than 0.05% of appendicitis cases [13]. The pain associated with appendicitis in such rare locations calls for various differential diagnoses, especially in females. Such pain could be linked to many gynecological-specific diseases such as pelvic inflammatory disease, tubo-ovarian abscess, endometriosis, ruptured or hemorrhagic ovarian cysts, adnexal torsion uterine fibroids presenting acutely with torsion, hemorrhage, or infarct, ectopic pregnancy, chorioamnionitis, retroverted gravid uterus, spontaneous or traumatic uterine rupture, and threatened, spontaneous abortion [14]. Therefore, appendicitis in rare locations can be misdiagnosed, as depicted in Table 1 [15].

**Table 1:** Possible misdiagnosis of appendicitis at rare locations

Appendicitis site	Common misdiagnosis
Ileum-crossing	Enteritis
Left lower quadrant	Diverticulitis
Periadnexal	Torsion of the ovary, extrauterine pregnancy, inflammation
Periduodenal	Duodenal ulcer, duodenitis
Perirenal	Pyelonephritis, renal colic
Scrotal hernia	Orchitis, torsion of the testis, incarceration
Subhepatic	Liver abscess, cholecystitis
Ureter-crossing	Hydronephrosis

While the incidence of appendicitis is relatively high, the variability in anatomical presentations and complications necessitates a nuanced approach to its diagnosis and treatment. As observed in this case, the atypical adherence of the appendix to the left ovary underscores the need for a thorough exploration during surgical interventions to uncover unexpected pathologies.

## Conclusions

This case report highlights the clinical significance of an uncommon anatomical adherence of the appendix to the left ovary, revealing a rare form of appendicitis. The successful laparoscopic intervention addressed this distinctive pathology and brought to light an incidental umbilical hernia, underscoring the importance of a thorough investigation during surgical procedures. This case underlines the necessity of a subtle diagnostic approach, especially in instances of unusual pain localization, and sheds light on the advantages of laparoscopic appendectomy in managing complex appendiceal variations. These findings contribute to the evolving comprehension of appendicitis presentations, offering valuable insights for clinicians in devising more personalized and efficient surgical strategies.
